# 5-(2-Chloro­phen­oxy)-1,3-dimethyl-1*H*-pyrazole-4-carbaldehyde oxime

**DOI:** 10.1107/S1600536812026530

**Published:** 2012-06-16

**Authors:** Hai-Jun Zhang, Chong-Guang Fan, Lei Shi

**Affiliations:** a College of Chemistry and Chemical Engineering, Nantong University, Nantong 226019, People’s Republic of China

## Abstract

In the title mol­ecule, C_12_H_12_ClN_3_O_2_, the benzene and pyrazole rings are inclined to each other at a dihedral angle of 83.3 (3)°. In the crystal, mol­ecules are linked into [010] chains *via* O—H⋯N hydrogen bonds with the unsubstituted pyrazole N atom acting as the acceptor.

## Related literature

For a related structure, see: Dai *et al.* (2011 ▶).
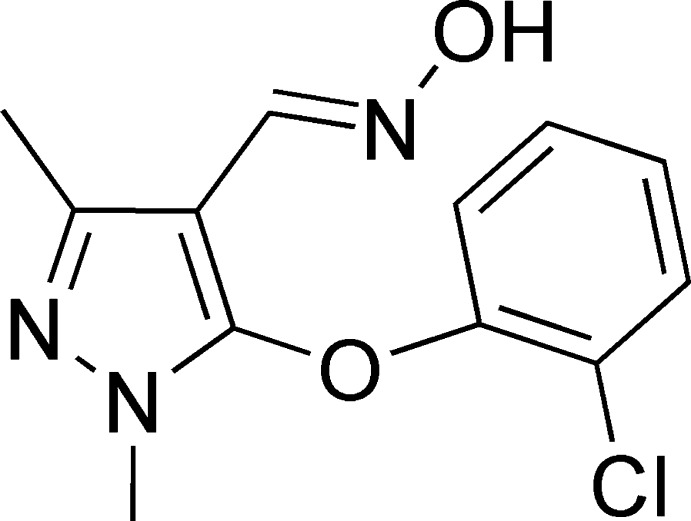


## Experimental

### 

#### Crystal data

C_12_H_12_ClN_3_O_2_
*M* *_r_* = 265.70
Monoclinic, 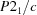

*a* = 11.108 (2) Å
*b* = 14.998 (3) Å
*c* = 8.0839 (16) Å
β = 104.94 (3)°
*V* = 1301.2 (4) Å^3^
*Z* = 4
Mo *K*α radiation
μ = 0.29 mm^−1^
*T* = 113 K
0.30 × 0.25 × 0.20 mm

#### Data collection

Rigaku SCXmini diffractometer
Absorption correction: multi-scan (*CrystalClear*; Rigaku, 2008 ▶) *T*_min_ = 0.918, *T*_max_ = 0.944
10731 measured reflections
2288 independent reflections
1638 reflections with *I* > 2σ(*I*)
*R*_int_ = 0.064

#### Refinement

*R*[*F* ^2^ > 2σ(*F* ^2^)] = 0.061
*wR*(*F* ^2^) = 0.159
*S* = 0.99
2288 reflections
166 parameters
H-atom parameters constrained
Δρ_max_ = 0.21 e Å^−3^
Δρ_min_ = −0.25 e Å^−3^

### 

Data collection: *CrystalClear* (Rigaku, 2008 ▶); cell refinement: *CrystalClear*; data reduction: *CrystalClear*; program(s) used to solve structure: *SHELXS97* (Sheldrick, 2008 ▶); program(s) used to refine structure: *SHELXL97* (Sheldrick, 2008 ▶); molecular graphics: *SHELXTL* (Sheldrick, 2008 ▶); software used to prepare material for publication: *SHELXTL*.

## Supplementary Material

Crystal structure: contains datablock(s) global, I. DOI: 10.1107/S1600536812026530/cv5311sup1.cif

Structure factors: contains datablock(s) I. DOI: 10.1107/S1600536812026530/cv5311Isup2.hkl

Supplementary material file. DOI: 10.1107/S1600536812026530/cv5311Isup3.cml

Additional supplementary materials:  crystallographic information; 3D view; checkCIF report

## Figures and Tables

**Table 1:** Hydrogen-bond geometry (Å, °)

*D*—H⋯*A*	*D*—H	H⋯*A*	*D*⋯*A*	*D*—H⋯*A*
O2—H2⋯N2^i^	0.82	1.97	2.787 (3)	171

Symmetry code: (i) 
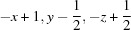
.

**Fig. 1.**
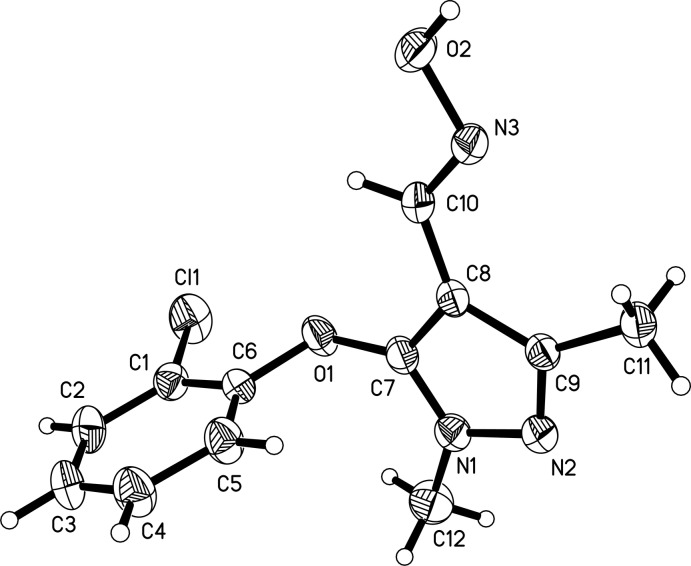
The molecular structure of (I) with displacement ellipsoids drawn at the 30% probability level.

## supplementary crystallographic information

### Comment

In a continuation of our search for new pyrazole oxime derivatives (Dai *et al.*, 2011), we present here the title compound, (I).

In (I) (Fig. 1), all bond lengths and angles are normal and comparable with those observed in the related compound (Dai *et al.*, 2011). The plane of substituted phenyl ring makes a dihedral angle of 83.3 (3)° with the pyrazole ring. In the crystal, intermolecular O—H···N hydrogen bonds (Table1) link molecules into chains in [010].

### Experimental

To a stirred solution of hydroxylamine hydrochloride (6 mmol) and potassium hydroxide (8 mmol) in methanol (30 ml) was added 5-(2-chlorophenoxy)-1,3-dimethyl-1*H*-pyrazole-4-carbaldehyde (4 mmol). The resulting mixture was heated to reflux for 6 h. The reaction mixture was cooled and poured into cold water (80 ml). The resulting colourless solid was collected and then recrystallized from ethyl acetate to give colourless crystals.

### Refinement

All H atoms were placed in calculated positions, with C–H = 0.93 and 0.96 ° A, and included in the final cycles of refinement using a riding model, with *U*_iso_(H) = 1.2–1.5 *U*_eq_(C).

### Crystal data

**Table:**

C_12_H_12_ClN_3_O_2_	*F*(000) = 552
*M**_r_* = 265.70	*D*_x_ = 1.356 Mg m^−^^3^
Monoclinic, *P*2_1_/*c*	Mo *K*α radiation, λ = 0.71073 Å
Hall symbol: -P 2ybc	Cell parameters from 10109 reflections
*a* = 11.108 (2) Å	θ = 3.1–27.7°
*b* = 14.998 (3) Å	µ = 0.29 mm^−^^1^
*c* = 8.0839 (16) Å	*T* = 113 K
β = 104.94 (3)°	Prism, colourless
*V* = 1301.2 (4) Å^3^	0.30 × 0.25 × 0.20 mm
*Z* = 4	

### Data collection

**Table:**

Rigaku SCXmini diffractometer	2288 independent reflections
Radiation source: fine-focus sealed tube	1638 reflections with *I* > 2σ(*I*)
Graphite monochromator	*R*_int_ = 0.064
ω and φ scans	θ_max_ = 25.0°, θ_min_ = 3.1°
Absorption correction: multi-scan (*CrystalClear*; Rigaku, 2008)	*h* = −13→13
*T*_min_ = 0.918, *T*_max_ = 0.944	*k* = −17→17
10731 measured reflections	*l* = −9→9

### Refinement

**Table:**

Refinement on *F*^2^	Primary atom site location: structure-invariant direct methods
Least-squares matrix: full	Secondary atom site location: difference Fourier map
*R*[*F*^2^ > 2σ(*F*^2^)] = 0.061	Hydrogen site location: inferred from neighbouring sites
*wR*(*F*^2^) = 0.159	H-atom parameters constrained
*S* = 0.99	*w* = 1/[σ^2^(*F*_o_^2^) + (0.0775*P*)^2^ + 0.5684*P*] where *P* = (*F*_o_^2^ + 2*F*_c_^2^)/3
2288 reflections	(Δ/σ)_max_ = 0.096
166 parameters	Δρ_max_ = 0.21 e Å^−^^3^
0 restraints	Δρ_min_ = −0.25 e Å^−^^3^

### Special details

**Table:**

Geometry. All e.s.d.'s (except the e.s.d. in the dihedral angle between two l.s. planes) are estimated using the full covariance matrix. The cell e.s.d.'s are taken into account individually in the estimation of e.s.d.'s in distances, angles and torsion angles; correlations between e.s.d.'s in cell parameters are only used when they are defined by crystal symmetry. An approximate (isotropic) treatment of cell e.s.d.'s is used for estimating e.s.d.'s involving l.s. planes.
Refinement. Refinement of *F*^2^ against ALL reflections. The weighted *R*-factor *wR* and goodness of fit *S* are based on *F*^2^, conventional *R*-factors *R* are based on *F*, with *F* set to zero for negative *F*^2^. The threshold expression of *F*^2^ > σ(*F*^2^) is used only for calculating *R*-factors(gt) *etc*. and is not relevant to the choice of reflections for refinement. *R*-factors based on *F*^2^ are statistically about twice as large as those based on *F*, and *R*- factors based on ALL data will be even larger.

### Fractional atomic coordinates and isotropic or equivalent isotropic displacement parameters (Å^2^)

**Table:**

	*x*	*y*	*z*	*U*_iso_*/*U*_eq_	
Cl1	0.95727 (9)	0.11946 (8)	−0.10002 (12)	0.0797 (4)	
O1	0.78065 (18)	0.08065 (15)	0.0915 (3)	0.0555 (6)	
N2	0.5175 (2)	0.13782 (17)	0.2347 (3)	0.0504 (7)	
N3	0.5840 (2)	−0.14771 (16)	0.2182 (4)	0.0526 (7)	
N1	0.6218 (2)	0.15655 (17)	0.1805 (3)	0.0502 (7)	
C7	0.6784 (3)	0.0805 (2)	0.1570 (4)	0.0459 (8)	
C10	0.6468 (3)	−0.0833 (2)	0.1825 (4)	0.0489 (8)	
H10	0.7180	−0.0960	0.1466	0.059*	
O2	0.6382 (2)	−0.22945 (15)	0.1961 (4)	0.0742 (8)	
H2	0.5990	−0.2700	0.2262	0.111*	
C6	0.8951 (3)	0.10481 (19)	0.2006 (4)	0.0437 (7)	
C1	0.9878 (3)	0.1260 (2)	0.1212 (4)	0.0488 (8)	
C8	0.6126 (2)	0.0091 (2)	0.1949 (4)	0.0418 (7)	
C9	0.5111 (3)	0.0494 (2)	0.2418 (4)	0.0421 (7)	
C5	0.9187 (3)	0.1079 (2)	0.3747 (4)	0.0585 (9)	
H5	0.8565	0.0936	0.4281	0.070*	
C2	1.1039 (3)	0.1516 (2)	0.2186 (5)	0.0618 (10)	
H2A	1.1660	0.1663	0.1651	0.074*	
C4	1.0363 (3)	0.1327 (3)	0.4714 (5)	0.0685 (10)	
H4	1.0533	0.1337	0.5903	0.082*	
C11	0.4042 (3)	0.0054 (2)	0.2915 (4)	0.0527 (8)	
H11A	0.3653	0.0475	0.3508	0.079*	
H11B	0.4344	−0.0444	0.3650	0.079*	
H11C	0.3444	−0.0150	0.1904	0.079*	
C3	1.1281 (3)	0.1556 (2)	0.3924 (5)	0.0649 (10)	
H3	1.2060	0.1737	0.4574	0.078*	
C12	0.6535 (4)	0.2479 (2)	0.1475 (5)	0.0698 (11)	
H12A	0.7002	0.2747	0.2523	0.105*	
H12B	0.5785	0.2813	0.1026	0.105*	
H12C	0.7027	0.2479	0.0658	0.105*	

### Atomic displacement parameters (Å^2^)

**Table:**

	*U*^11^	*U*^22^	*U*^33^	*U*^12^	*U*^13^	*U*^23^
Cl1	0.0725 (7)	0.1143 (9)	0.0598 (6)	−0.0115 (6)	0.0303 (5)	0.0005 (5)
O1	0.0435 (12)	0.0732 (15)	0.0515 (13)	−0.0175 (11)	0.0154 (10)	−0.0114 (12)
N2	0.0440 (15)	0.0478 (17)	0.0599 (17)	−0.0044 (12)	0.0146 (13)	−0.0034 (12)
N3	0.0401 (14)	0.0422 (15)	0.076 (2)	0.0033 (12)	0.0152 (13)	0.0009 (13)
N1	0.0462 (15)	0.0455 (16)	0.0596 (18)	−0.0065 (13)	0.0151 (13)	−0.0003 (13)
C7	0.0357 (15)	0.054 (2)	0.0471 (18)	−0.0056 (15)	0.0090 (13)	−0.0038 (15)
C10	0.0344 (15)	0.052 (2)	0.062 (2)	−0.0015 (15)	0.0148 (14)	−0.0025 (16)
O2	0.0556 (14)	0.0429 (14)	0.133 (2)	0.0071 (11)	0.0413 (15)	0.0007 (15)
C6	0.0377 (16)	0.0433 (18)	0.0497 (19)	−0.0055 (13)	0.0106 (14)	−0.0037 (14)
C1	0.0454 (18)	0.0468 (18)	0.056 (2)	−0.0005 (14)	0.0155 (15)	0.0048 (15)
C8	0.0326 (14)	0.0462 (18)	0.0449 (18)	−0.0034 (13)	0.0070 (13)	−0.0017 (14)
C9	0.0363 (15)	0.0460 (18)	0.0424 (17)	−0.0036 (13)	0.0072 (13)	−0.0028 (14)
C5	0.0478 (19)	0.076 (2)	0.056 (2)	−0.0112 (17)	0.0200 (16)	−0.0001 (18)
C2	0.0400 (18)	0.074 (2)	0.074 (3)	−0.0041 (17)	0.0190 (17)	0.009 (2)
C4	0.057 (2)	0.092 (3)	0.050 (2)	−0.005 (2)	0.0039 (17)	0.0002 (19)
C11	0.0390 (16)	0.063 (2)	0.057 (2)	−0.0041 (15)	0.0141 (14)	−0.0010 (17)
C3	0.0366 (18)	0.081 (3)	0.073 (3)	−0.0074 (17)	0.0067 (17)	0.002 (2)
C12	0.073 (2)	0.052 (2)	0.087 (3)	−0.0079 (18)	0.026 (2)	0.0095 (19)

### Geometric parameters (Å, º)

**Table:**

Cl1—C1	1.735 (3)	C8—C9	1.415 (4)
O1—C7	1.371 (3)	C9—C11	1.501 (4)
O1—C6	1.396 (3)	C5—C4	1.389 (5)
N2—C9	1.330 (4)	C5—H5	0.9300
N2—N1	1.369 (3)	C2—C3	1.362 (5)
N3—C10	1.267 (4)	C2—H2A	0.9300
N3—O2	1.397 (3)	C4—C3	1.379 (5)
N1—C7	1.339 (4)	C4—H4	0.9300
N1—C12	1.456 (4)	C11—H11A	0.9600
C7—C8	1.376 (4)	C11—H11B	0.9600
C10—C8	1.446 (4)	C11—H11C	0.9600
C10—H10	0.9300	C3—H3	0.9300
O2—H2	0.8200	C12—H12A	0.9600
C6—C5	1.364 (4)	C12—H12B	0.9600
C6—C1	1.383 (4)	C12—H12C	0.9600
C1—C2	1.381 (5)		
			
C7—O1—C6	117.8 (2)	C6—C5—C4	119.5 (3)
C9—N2—N1	106.1 (2)	C6—C5—H5	120.2
C10—N3—O2	111.1 (3)	C4—C5—H5	120.2
C7—N1—N2	109.8 (2)	C3—C2—C1	120.5 (3)
C7—N1—C12	129.1 (3)	C3—C2—H2A	119.8
N2—N1—C12	121.1 (3)	C1—C2—H2A	119.8
N1—C7—O1	121.3 (3)	C3—C4—C5	120.4 (3)
N1—C7—C8	109.6 (3)	C3—C4—H4	119.8
O1—C7—C8	128.9 (3)	C5—C4—H4	119.8
N3—C10—C8	123.0 (3)	C9—C11—H11A	109.5
N3—C10—H10	118.5	C9—C11—H11B	109.5
C8—C10—H10	118.5	H11A—C11—H11B	109.5
N3—O2—H2	109.5	C9—C11—H11C	109.5
C5—C6—C1	120.2 (3)	H11A—C11—H11C	109.5
C5—C6—O1	124.2 (3)	H11B—C11—H11C	109.5
C1—C6—O1	115.6 (3)	C2—C3—C4	119.7 (3)
C6—C1—C2	119.8 (3)	C2—C3—H3	120.2
C6—C1—Cl1	119.7 (2)	C4—C3—H3	120.2
C2—C1—Cl1	120.6 (3)	N1—C12—H12A	109.5
C7—C8—C9	103.5 (3)	N1—C12—H12B	109.5
C7—C8—C10	124.5 (3)	H12A—C12—H12B	109.5
C9—C8—C10	132.0 (3)	N1—C12—H12C	109.5
N2—C9—C8	111.0 (3)	H12A—C12—H12C	109.5
N2—C9—C11	120.3 (3)	H12B—C12—H12C	109.5
C8—C9—C11	128.6 (3)		

### Hydrogen-bond geometry (Å, º)

**Table:**

*D*—H···*A*	*D*—H	H···*A*	*D*···*A*	*D*—H···*A*
O2—H2···N2^i^	0.82	1.97	2.787 (3)	171

Symmetry code: (i) −*x*+1, *y*−1/2, −*z*+1/2.
